# Editorials

**Published:** 1908-08

**Authors:** 


### EDITORIALS

**The Business Office of the JOURNAL-RECORD is 1 1-2 to 5 1-2 South Broad St.**
**The Editorial Office is 1014-15 Century Bldg.**
**Address all Business Communications to Journal-Record of Medicine, 1 1-2 to 5 1-2 South Broad Street.**
**Make remittances payable to THE JOURNAL-RECORD OF MEDICINE.**
**On matters pertaining to the Editorial and Original communications, address Edgar G. Ballenger, M. D., Atlanta, Ga.**
**Reprints of original articles will be furnished at cost price. Requests for the same should always be made in THE MANUSCRIPT.**
**We will present, postpaid, on request, to each contributor of an original article, twenty (20) marked copies of THE JOURNAL-RECORD OF MEDICINE containing such article.**

### TO COMBAT FLIES BY REMOVING THEIR BREEDING PLACES.

We desire to commend most heartily the effort of Dr. Claude Smith to have the city council pass an ordinance placing a tax of $2 on all horses and cows kept within the city limits. Practically all house flies are bred in stables, consequently it is a simple matter to exterminate them by a thorough and concerted action. The house fly never goes far from the place where it was hatched. Each citizen should therefore look out for his own premises.

The expenditure of $15,000 to $18,000 will be required to remove the manure from the stables of Atlanta, and it is thought by the chief of the sanitary department that a tax of $2 for horses and cows will provide this sum.

The resolution to be introduced in council, which was gotten up by Dr. Claude A. Smith, the city bacteriologist, is along the following lines: It shall be unlawful for any person owning stables, pens, stalls, to allow them to become filthy or unwholesome. Persons owning cows, horses and mules shall be taxed $2 per head per year. Garbage will be required to be kept in watertight vessels, thus preventing flies. Manure shall be placed in bins or pits and covered until removed. Penalty for the violation of either one of these will be not less than $1 or more than $100.

The National Government is also to prosecute a systematic campaign against house flies and musquitoes, and thus will give great encouragement to the efforts of local boards of health to exterminate these annoying and disease-producing insects.

### SPECIAL NUMBER OF THE PRACTITIONER ON THE OPSONIC METHOD AND VACCINE THERAPY.

The May issue of the Practitioner, London, is devoted entirely to a consideration of the opsonic method and vaccine therapy. This excellent Journal occasionally publishes a special number upon one important subject and we think the present discussion exceedingly interesting and opportune.

Vaccine therapy opens an entirely new field in medicine and one in which there is opportunity either for an infinite amount of good or harm according as the "immunisator," as Wright has appropriately designated him, is equiped for this form of treatment.

Editorially, the Practitioner takes conservative grounds and states that the enthusiast may believe that this method forms a panacea for all the infectious diseases, while another may deny that it is of the slightest value; the truth, no doubt, lies between these two extremes. It should be tried in those cases in which its use does not interfere in any way with the employment of other methods; but in those cases where surgical means are indicated, and are likely to be efficacious, there should be no delay in order to employ vaccine-therapy.

A word of warning is given to the physician unfamiliar with all of the facts, to prevent the occurrence of another fiasco similar to that which followed the introduction of tuberculin.

The first paper in this number is by Sir Almroth E. Wright, on "Some Points in Connection with Vaccine-Therapy and Therapeutic Immunisation Generally." Considerable space is devoted to the question of whether or not the opsonic index is necessary in the application of this treatment. Although admitting that when the clinical symptoms are of such a nature as to give the physician a reasonably good idea of the progress being made, satisfactory results may be obtained, as a rule, he thinks that the administration of vaccones should be regulated by the opsonic method. Much emphasis is placed upon the consideration of bringing the antibacterial agencies of the circulating blood into effective operation upon the microbes in a radius of infection.

Wells reports some "Observations on the Opsonic Index in the Infant," and reaches the conclusion that the anti-bacterial defense of the child is not dependent upon the opsonic contents of the serum.

Harris thinks, from his own experience and from corroborative evidence of other investigators, that this treatment will be recognized eventually as one of distinct value in pneumonia.

He has found that rest after inoculation is necessary for a good opsonic upward flow; active exercise on the other hand produces a sharp negative phase.

The surgeon is urged to observe the opsonic index before certain operations, especially in tubercle, and if it is low, and, time will permit, to raise it before and not after the operation if we wish to obtain rapid heating.

Inman presents an article on "The Value of the Opsonic Index in the Treatment of Pulmonary Tuberculosis," and has found that in the patient with advanced pulmonary tuberculosis the opsonic index varies widely from day to day and without apparent rhyme or reason. This is due to irregular spontaneous anto-inoculation from the focus in the lungs where the bacilli are multiplying. In such cases rest is of prime importance. Another factor of moment is to increase the co-agulability of the blood by the exhibition of calcium salts. A milk diet tends to produce the same effect and he thinks this is one of the unrecognized advantages secured by the extensive use of milk in the treatment of tuberculosis. The results of vaccines in pulmonary tuberculosis are probably less satisfactory than in other conditions. Inman summarizes his paper as follows: Early or febrile cases of pulmonary tuberculosis may be treated with advantage by means of pure air and graduated exercises. When such treatment is undertaken it must be borne in mind that "tuberculin by anto-inoculation is being used." The opsonic index is a valuable guide to such treatment, and also gives useful information if inoculations of Koch's tuberculin are employed. Rest is essential in febrile cases of consumption. and in these cases, injections of tuberculin, using as a guide the opsonic index, is the treatment indicated."

---

### BILL REQUIRING MEDICAL EXAMINATION BEFORE OBTAINING MARRIAGE LICENSE.

At the regular meeting of the Fulton County Medical Society, held July 2, the Hon. L. A. Dean, of the Georgia State Legislature, addressed the society and asked its support for a bill he had introduced this present session.

We deem this of importance to all practitioners and so present the bill for consideration.

A BILL TO BE ENTITLED

An act to prevent the issuing of marriage license to persons having the disease of gonorrhoea or syphilis in communicable stages and to regulate and prescribe the manner of issuing marriage license and to provide a punishment and declare the effect on the marriage contract, for the violation of the terms of this act, and for other purposes.

SECTION 1. Be it enacted by the General Assembly of the State of Georgia and it is hereby enacted by authority of the same: That from and after the passage of this act it shall be unlawful for any ordinary of this State to issue a marriage license until the male person applying therefor has made an affidavit signed and sworn to before the ordinary stating that within ten days prior to the date of the affidavit he has submitted himself to a thorough examination by a competent and reputable physician and based upon such examination and upon his own knowledge of himself he verily believes he is not the subject of acute or latent gonorrhoea or syphilis in a communicable stage, and that he has not been treated for gonorrhoea in such stage within six months, nor for syphilis within twelve months from the date of the affidavit; and shall also file a certificate made by a licensed physician of some school authorized to practice medicine and surgery in this State, stating that he has within ten days prior to the date of the certificate examined the applicant, and that such examination was made with due skill and knowledge and with sufficient time to determine whether such person is a subject of acute or latent gonorrhoea or syphilis in a communicable stage, that his professional judgment is satisfied that such is not subject to any such disease in a communicable stage, and that on professional honor and integrity he gives the certificate with a full realization of the suffering entailed upon the wife and offspring by marriage with persons having such disease. Said affidavit and certificate to be made not more than twenty days prior to the date of issuing the license.

SEC. 2. Be it further enacted, That said affidavit and said certificate being filed with the ordinary, he may issue a marriage license to the applicant provided he is otherwise qualified to marry under existing laws; which license shall be and remain of force and authorize the marriage of the applicant only for a period of twenty days after the date thereof.

SEC. 3. Be it further enacted, That the making of a false affidavit by the applicant for license shall be ground for total divorce at the instance of the wife, provided suit therefor is brought within two years after the date of the marriage.

SEC. 4. Be it further enacted, That the making of a false certificate on the part of a physician shall be a misdemeanor, and on conviction such physician shall be punished as for a misdemeanor. That the making of a false affidavit by the applicant for license shall be false swearing and on conviction be punished as by law provided for such offense.

SEC. 5. Be it further enacted, That all laws and parts of laws in conflict herewith be, and they are hereby repealed.

The discussion following the reading of the bill was general and brought out many points. The chief of these was that the end most to be desired would not be reached by provisions presented. The general practitioner is not competent to say positively when a man is free from communicable gonorrhoea. The methods of testing for this disease are so complex and require such accurate technic that none but the specialist can be secure in his conclusions regarding a questionable case. All agreed as to this, and Mr. Dean said that the question of a Board of Examiners had been raised in the committee only to be dropped because whatever of complexity was placed in the bill would tend to defeat it and prevent any start whatsoever along this line from being made at this time.

The question of time limits arose and the members were unanimous in saying six months was too short a period after gonorrhoea to be safe.

The paragraph regarding divorce caused a brief discussion, but Mr. Dean stated that this had the approval of several clergymen, who, while they hesitated to approve of divorce on any ground, felt that the prevention of a perpetuation of a curse among the generations was the best of reasons for separation.

Finally the Society approved of the bill as it stands and agreed to make every effort to secure its passage, realizing that thereby a good foothold would be secured for further advancement scientifically and that there might be a basis for educational propaganda among the people. It was particularly noticeable that the discussion turned almost entirely upon gonorrhoea and its dangers rather than upon syphilis. The horrid importance of this disease is well known to the profession, but the laity rests in grievous ignorance of its fearful menace.

It was resolved that the Society give its unqualified approval to the bill, that members do all they could personally to secure its passage; that the various county societies of the State, be communicated with and urged to support it, and that a copy of the resolution be given to the newspapers for publication.

A vote of thanks was tendered the Hon. L. A. Dean for his efforts in behalf of hygiene and for presenting the matter to the Society.

### SOUTHERN MEDICAL ASSOCIATION.

The next annual meeting of the Southern Medical Association will be held in Atlanta, November 10, 11 and 12, 1908, and it may be confidently predicted that this will be one of the most successful meetings of this representative and growing association. The last meeting was held in Birmingham, Ala., where the following officers were elected: President, B. L. Wyman, Birmingham, Ala.; Vice-Presidents, W. P. McAdory, Birmingham, Ala.; H. M. Folkes, Biloxi, Miss.; Frank Watson, New Orleans, La.; G. R. Holden, Jacksonville, Fla.; Raymond Wallace, Chattanooga, Tenn.; A. L. Fowler, Atlanta, Ga. Secretary-Treasurer, Oscar Dowling, Shreveport, La. Counsellors, D. F. Talley, Birmingham, Ala.; Michael Hoke, Atlanta, Ga.; John M. McDiarmid, DeLand, Fla.; W. W. Crawford, Hattiesburg, Miss.; W. W. Butterworth, New Orleans, La.; Geo. C. Savage, Nashville, Tenn. Section on Medicine, Chairman, Seale Harris, Mobile, Ala.; Secretary, H. E. Mitchell, Birmingham, Ala. Section on Surgery, Chairman, W. F. Westmoreland, Atlanta, Ga.; Secretary, J. L. Crook, Jackson, Tenn. Section on Opthalmology, Chairman, J. F. Herron, Jackson, Tenn.; Secretary, A. B. Harris, Birmingham, Ala.

